# Variation in electrosurgical vessel seal quality along the length of a porcine carotid artery

**DOI:** 10.1177/0954411915621092

**Published:** 2016-01-07

**Authors:** Hayley Louise Wyatt, Rosie Richards, Rhys Pullin, TH Jimmy Yang, Emma J Blain, Sam L Evans

**Affiliations:** 1 Cardiff School of Engineering, Cardiff University, Cardiff, UK; 2 Gyrus Medical Ltd, Cardiff, UK; 3 Cardiff School of Biosciences, Cardiff University, Cardiff, UK

**Keywords:** Electrosurgery, blood vessels, morphology, vessel sealing, bipolar

## Abstract

Electrosurgical vessel sealing has been demonstrated to have benefits for both patients and practitioners, but significant variation in the strength of the seal continues to be a concern. This study aims to examine the variation in electrosurgical seal quality along the length of a porcine common carotid artery and explore the relationships between seal quality, vessel size and morphology. Additionally, the study aimed to investigate the minimum safety threshold for successful seals and the influence of vessel characteristics on meeting this requirement. A total of 35 porcine carotid arteries were sealed using the PlasmaKinetic Open Seal device (Gyrus). Each seal was burst pressure tested and a sample taken for staining with elastin van Gieson’s stain, with morphological quantification using image processing software ImageJ. With increasing distance from the bifurcation, there was an increase in seal strength and a reduction in both elastin content and vessel outer diameter. A significant correlation was found between burst pressure with both outer diameter (p < 0.0001) and elastin content (p = 0.001). When considering the safe limits of operation, vessels of less than 5 mm in outer diameter were shown to consistently produce a seal of a sufficient strength (burst pressure > 360 mmHg) irrespective of vessel morphology.

## Introduction

Electrosurgical blood vessel sealing devices are used in a variety of surgical applications including both laparoscopic and open surgery and have been demonstrated to reduce both operative time and costs and lead to less post-operative pain for the patient.^1,2^ Such devices work by passing a high-frequency alternating current through the tissue, causing the tissue to heat up and collagen and other cellular proteins to shrink and denature.^3,4^ Although the benefits of such devices have been widely reported in the literature, there is significant variation in the quality of the seal achieved, with reported burst pressures (BPs) varying between approximately 170 and 1934 mmHg.^3,5–7^

Previous studies have explored different factors affecting the quality of the seal, including the force and temperature applied to the tissue by the electrosurgical device when creating the seal. For both force and temperature, a minimum and maximum threshold exists; below the minimum threshold, a strong seal will not be created, whereas above the maximum threshold, the application of excessive heat and force can cause tissue damage and weaken the resulting seal.^8^ Furthermore, the properties of the vessel were also found to affect the seal quality. For example, with increasing outer diameter, there was a progressive reduction in the burst strength of the seal.^5^ More recently, the effect of vessel morphology on seal quality has been investigated with the collagen–elastin ratio providing a good prediction of seal strength when considering vessels from different regions of the body.^7^ The effect of morphology on seal quality has been discussed in a number of studies as a possible explanation for the different behaviours of arteries and veins and highlights the importance of morphology in the sealing process.^6,8^

Morphology of the vessel varies depending on anatomical location, age, sex, species and vessel size. Furthermore, the morphology of a vessel can vary significantly along its length. For example, the proximal region (the region closest to the heart) of a porcine common carotid artery has a greater elastin content than the distal regions, with a gradual reduction in elastin content along the length of the artery. Additionally, as the level of elastin decreases, there is an increase in the smooth muscle cells but little change in the level of collagen.^8–10^

The aim of this study was to examine the variation in electrosurgical seal quality along the length of a porcine common carotid artery and to explore the relationships between seal quality, vessel size and vessel morphology. Additionally, the study aimed to investigate the minimum safety threshold for successful seals and the influence of vessel characteristics on meeting this requirement. It is hoped that knowledge attained from the study can be used to improve device performance and thus seal quality by providing a greater understanding of the effects of vessel characteristics.

## Materials and method

### Tissue preparation

**Figure 1.**
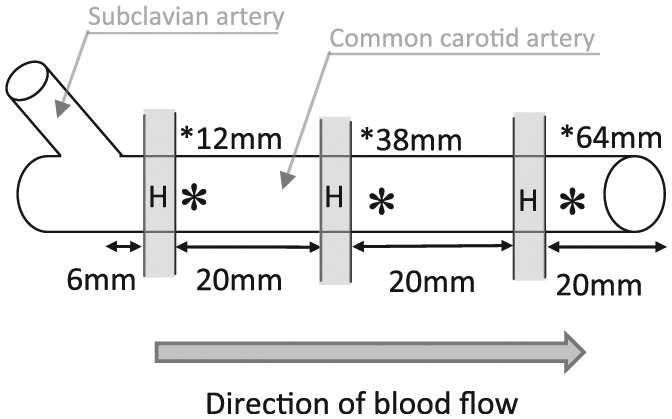
Schematic illustration of the sectioning of a blood vessel. * indicates the location of the electrosurgical seal, and H shows the position of the 2-mm ring sample taken for histology.

Porcine common carotid arteries, from pigs aged 4–6 months, were obtained from a local abattoir, with all testing conducted within 10 h of slaughter. Vessels were skeletonised and sectioned (Figure 1) using a scalpel; a 2-mm sample was fixed in 10% buffered formalin for histological analysis and a 20-mm section taken for sealing. The position from the bifurcation was measured, along with the outer vessel diameter and wall thickness. All measurements were taken after the vessels were sectioned using digital Vernier callipers. The outer diameter of the vessel being measured without compression or internal pressure, each measurement was repeated three times. The vessel wall thickness was measured by placing one side of the callipers within the vessel lumen and the other side below, taking care not to compress the vessel wall, resulting in the measurement of the wall thickness; three measurements were taken at random locations around the vessel circumference.^11^

### Electrosurgical seal and inflation test

Vessel segments were then sealed at the section end (indicated by * in Figure 1) using the bipolar PlasmaKinetic Open Seal device (Gyrus) connected to a G400 generator using the default generator settings of VP2 60. The PlasmaKinetic Open Seal device is an electrosurgical forceps device, where the blood vessel was placed between the device jaws, and high-frequency alternating current was delivered to the blood vessel causing the collagen within the wall to denature and through the application of pressure form the seal. The vessel segment was placed between the device jaws and sealed in an unloaded state prior to connecting the vessel to the saline perfusion apparatus. The coagulation process was deemed to be complete based on the generator tone change as per the manufacturers’ instructions. The tone change is controlled by the continuous monitoring of tissue impedance through a feedback loop of the generator, thus producing each seal in a consistent manner.

**Figure 2.**
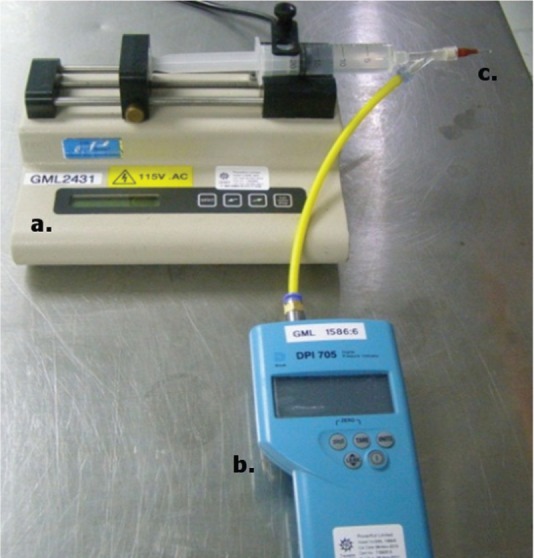
Diagrammatic representation of the saline perfusion set-up used for burst pressure testing sealed vessels: (a) Cole-Parmer syringe pump set at an infusion rate of 50 mL/h, (b) Druck digital pressure indicator and (c) blunt needle for the attachment of the blood vessel.

The perfusion apparatus was set up prior to sealing (Figure 2). The set-up consists of a syringe pump used to deliver saline and a digital pressure indicator (Druck) to measure the BP. A syringe was fixed into the syringe pump and connected to a three-point adapter. The remaining two outlets of the adapter were connected to the pressure indicator and a blunt needle used for specimen attachment. Saline was infused through the apparatus to ensure all air was removed.

The sealed carotid artery was then connected to the blunt needle using a haemostat, with the jaws covered with rubber to create a secure connection, and infused with physiological saline (10% w/v) at a rate of 50 mL/h, with saline at room temperature (20 °C). The BP, defined as a sudden and rapid decrease in seal pressure, was recorded for each segment. A seal was considered successful if it withstood a pressure greater than 360 mmHg; this pressure was chosen in line with standards used by Gyrus Medical Ltd and was based upon a value of three times the maximum systolic pressure, thus including a sufficient safety factor.

### Histology

**Figure 3.**
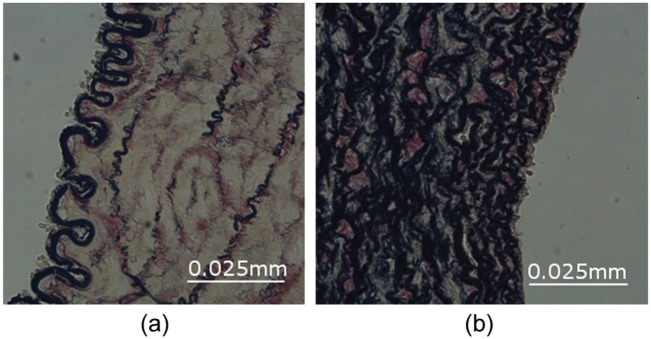
Representative sections depicting blood vessels stained with elastin van Gieson’s stain (elastin stained black, collagen stained red). Depiction of (a) a predominantly muscular vessel containing <20% elastin and (b) a predominantly elastic vessel containing ≥20% elastin (scale bar = 0.02 mm).

The tissue samples were processed for histological analysis. A total of 35 samples cut in the transverse plane were taken from nine different vessels for analysis. Using elastin van Gieson’s (EVG) stain, the elastin component of the vessels was stained black and the collagen component red (Figure 3). Images were collected using the Leica DMRB microscope with a Colour Moticam 2000 digital camera and basic image acquisition software (Motic Image Plus). Quantitative morphological analysis was carried out using a thresholding technique in image processing software ImageJ, with both collagen and elastin content quantified for each sample. Images were first converted to a greyscale image and then imported into ImageJ. A manual thresholding technique was then applied, with the threshold values adjusted until the area of interest was highlighted in red, the software then presented this highlighted region as a percentage area. The process was repeated for both elastin and collagen, with different threshold values applied based upon the morphological component of interest. Each measurement was repeated three times, and an average value was taken for the media layer of the vessel. It should be noted that the results from the image processing can depend greatly on the staining process and slice preparation; to reduce this effect, each image was analysed individually instead of using a batch processing technique.

### Statistical analysis

Data were tested for normality and equal variance using SPSS; as the data were not normally distributed, non-parametric tests were used. All data were used to test for significant correlations between the different variables with Spearman’s rank correlation test. A p-value of <0.05 rejected the null hypothesis that there was no correlation and indicated a significant relationship. An s value approaching −1 or +1 indicated a strong correlation with the sign indicating the direction.^12^

Data were grouped according to whether the segment was predominantly muscular or elastic (Figure 3), with muscular segments having an elastin content of less than 20% and a well-defined internal elastic lamina (IEL) and elastic segments having an elastin content of greater than or equal to 20% and no well-defined IEL. Differences between groups were tested using the Mann–Whitney test (two tailed), with a p-value of <0.05 rejecting the null hypothesis that the medians of the two groups were equal and indicating a significant difference between the two groups.^12^

## Results

Analysis of BP revealed a high variation with a range of 4–2285 mmHg. Vessels were grouped based on the position from the bifurcation, and data were analysed for BP, outer diameter, vessel thickness, elastin content and collagen content (Table 1).

**Table 1.** Data for vessels grouped according to the position from the bifurcation.

	12 mm	38 mm	64 mm	90 mm
Burst pressure (mmHg)	246.67 ± 193.00	963.22 ± 718.20	1076.11 ± 532.89	1243.38 ± 424.08
Outer diameter (mm)	5.91 ± 0.46	4.51 ± 0.95	3.50 ± 0.55	3.46 ± 0.44
Thickness (mm)	1.04 ± 0.26	0.86 ± 0.17	0.95 ± 0.15	0.92 ± 0.16
% Elastin	35.28 ± 5.21	26.25 ± 11.52	14.99 ± 8.95	10.95 ± 4.70
% Collagen	20.64 ± 2.11	22.18 ± 2.62	22.50 ± 3.04	21.54 ± 2.76

% Composition quantified in conjunction with ImageJ software. All data are presented as mean ± SD.

**Figure 4.**
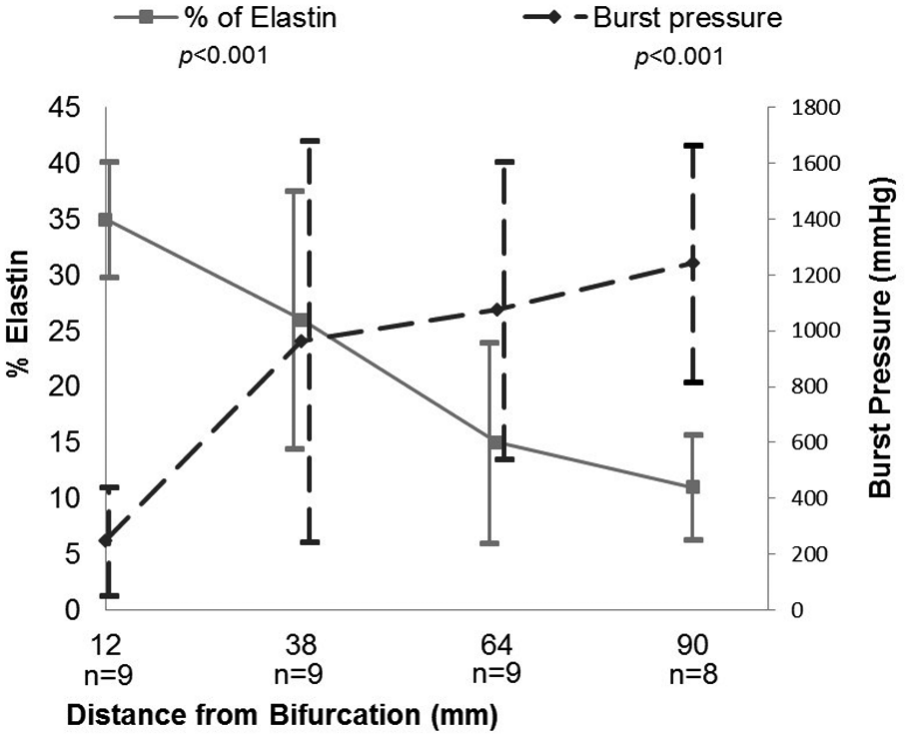
Variation in elastin content and burst pressure with position from bifurcation, % composition quantified in conjunction with ImageJ software. Data are presented as mean ± SD.

**Figure 5.**
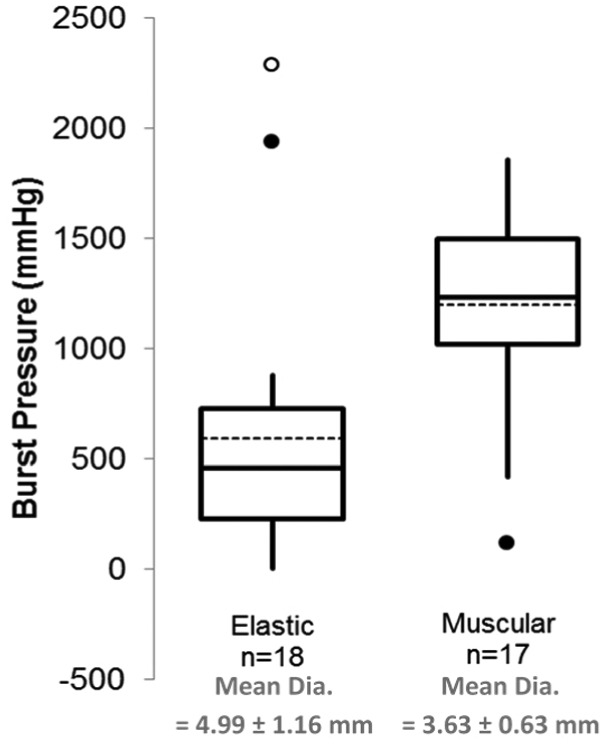
Box plot showing the difference in burst pressure with predominantly muscular and elastic vessels. The black circles show moderate outliers and white circles show extreme outliers.

A significant correlation was found between BP with both outer diameter (p ≤ 0.0001, s = −0.665) and % elastin (p = 0.001, s = −0.520; Figure 4). Furthermore, a correlation was also found between elastin and outer diameter (p ≤ 0.0001, s = 0.559). The results were further analysed by grouping the vessels as either predominantly muscular or predominantly elastic based on criteria described previously (Figure 3). When comparing the BP of the two groups, a significant difference was found (p = 0.001, Figure 5).

**Figure 6.**
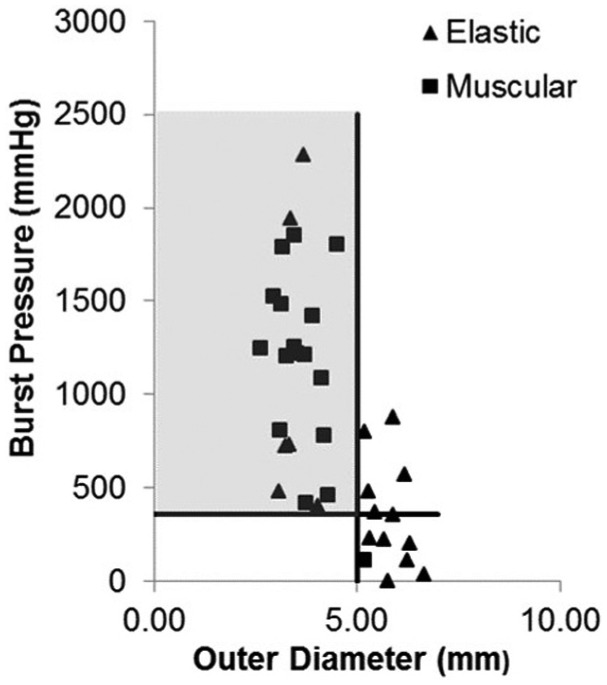
Scatter graph showing the relationship between vessel outer diameter and burst pressure (p ≤ 0.0001, s = −0.665), with data grouped as predominantly muscular or elastic based on the criteria described in Figure 3. Line A indicates the minimum standard for a sufficient seal, 360 mmHg, and line B indicates the maximum threshold for vessel size, 5 mm outer diameter.

Outliers were observed for the BP measurements (Figure 5), which were either considered as moderate (black circles) or extreme (white circles). The outliers are values which are beyond a range calculated using the first and third quartiles.^12^ It is possible to explain the outlier for the muscular group as this is the only muscular vessel with an outer diameter greater than 5 mm, and as shown in Figure 6, vessels with a diameter of greater than 5 mm are more likely to produce a weak seal. However, vessel size does not account for the outliers of the elastic group, and at present, there is no clear explanation for this.

The minimum threshold of a successful seal was observed, with a BP of 360 mmHg considered to be the minimum pressure (Figure 6), in line with standards used by Gyrus Medical Ltd. Using this as the minimum standard, it is possible to determine the maximum vessel size that will lead to a seal quality consistently meeting these standards. The vertical line B (Figure 6) shows that all vessels below 5 mm in outer diameter produce a seal above the minimum standard, irrespective of the morphology of the vessel. Furthermore, a relationship exists between vessel outer diameter and seal strength (Figure 6), with a reduction in outer diameter resulting in an increase in seal strength as mentioned previously.

## Discussion

Electrosurgical seal strength has previously been shown to vary based on vessel outer diameter, the region of the body the vessel is from and whether it is an artery or vein.^5–8^ This study investigated the variation in seal strength along the length of a porcine carotid artery, where the strength was found to increase with increasing distance from the bifurcation. Additionally, with increasing distance from the bifurcation, there was a reduction in elastin content and outer diameter of the vessel, suggesting that either of these factors may influence the seal quality. At 12 mm from the bifurcation, the lowest seal strength (246.67 ± 193.00 mmHg) was recorded, with this position also showing the least variation in seal strength. This could be due to the region of the vessel having little variation in both outer diameter (5.91 ± 0.46 mm) and elastin content (35.28% ± 5.21%). In the mid region of the vessels, at a distance of approximately 38 mm from the bifurcation, there was a large variation in seal strength (963.22 ± 718.20 mmHg), with this region having the highest variation in both elastin content (26.25% ± 11.52%) and vessel outer diameter (4.51 ± 0.95 mm).

With increasing distance from the bifurcation, there was a reduction in the amount of elastin with little change in the collagen content, supporting work by García et al.^9^ As a result, no correlation was found between collagen and any other variable. However, a significant correlation was found between seal strength and elastin content (p = 0.001, s = −0.520); with a reduction in elastin content, there was an increase in seal strength. Due to the histological staining used, it was not possible to quantify the smooth muscle content of the blood vessels, although an increase in smooth muscle cells with increasing distance from the bifurcation has been reported.^9^ Previous studies highlighted the importance of morphology on the seal strength,^6,8^ and it is thought that a reduction in smooth muscle content in more elastic vessels results in less resistance to thermal damage, possibly leading to larger areas being excessively heated,^6^ with excessive heating thought to damage the seal and tissue adjacent to the seal leading to a reduction in seal quality. This study found a significant difference between the BPs of predominantly muscular and elastic vessels (p = 0.0001), suggesting that electrosurgical sealing will be more effective in the more muscular regions of the blood vessels.

With increasing distance from the bifurcation, there was also a reduction in the outer diameter of the vessel, with this reduction in vessel size correlating with an increase in seal strength (p ≤ 0.0001, s = −0.665). This relationship has been widely reported in the literature^5,13^ with one explanation for this relationship being the increased amount of material between the device jaws for larger vessels leading to a more superficial penetration of current.^14^ This results in the greatest thermal effects being in the adventia layer of the blood vessel wall,^14^ although further work is required to explore this idea further. One possible solution to aid deeper penetration of current and a more even thermal spread throughout the vessel wall is to investigate the effects of the design of the contact surface of the device jaws. The jaws used throughout this study were flat with a roughened surface; other studies have found surfaces with varying structures, such as grooves, to have an effect on the strength of the seal and the temperature during the sealing process.^14–16^ Another suggestion would be the use of fine teeth of varying heights, similar to the pattern found on standard surgical forceps, to create a deeper penetration of the current and a more even thermal effect, although this would warrant significant investigation as it could induce damage to the tissue and have a detrimental effect on seal quality. It is thought that more research into the surface characteristics of the contact surface could lead to an improved seal strength and overall device performance.

A significant correlation existed between vessel outer diameter and elastin content (p ≤ 0.0001, s = 0.559); with a reduction in vessel outer diameter, there was a reduction in elastin content. This relationship makes it difficult to attribute the variation in seal strength to one factor alone, although it is thought that both vessel morphology and size do contribute significantly to seal strength. Although it is difficult to attribute an increase in seal strength to one factor, our data indicate that the size of the vessel is the most influential factor in determining the strength of a seal, with all vessels with an outer diameter of less than 5 mm resulting in a seal quality of sufficient standard (BP ≥ 360 mmHg), irrespective of whether the vessel was predominantly muscular or elastic. Additionally, the anomaly observed in the muscular group of our study can be explained by the fact that this was the only muscular sample with a vessel size greater than 5 mm, further demonstrating the significance of vessel size. However, vessel size does not explain the anomalies in the elastic group.

As stated previously, our results demonstrate that a seal of sufficient strength will be produced for a vessel with an outer diameter less than 5 mm. When looking at a porcine carotid artery, seals performed at a distance of greater than 64 mm from the bifurcation will also meet the minimum safety threshold, due to vessels of 5 mm or less being found in this region. Additionally, at this distance, the vessel morphology is more muscular (lower percentage of elastin) which is thought to help achieve a good seal strength. Vessels sealed at 12 mm from the bifurcation will be too large and elastic to produce a successful seal consistently. In the mid region of the vessel, approximately 38 mm from the bifurcation, there is large variation in the vessel diameter and vessel morphology, making it difficult to achieve a consistent seal quality in this region.

Although the trends do exist in the data, there is still substantial scatter, a finding that appears throughout a number of other studies.^3,5,7,8^ The change in vessel diameter and morphology does not appear to account for all the variation in the seal strength, and thus, further work is required to gain a greater understanding of the sealing process. However, the variation in seal strength can in part be attributed to varying vessel characteristics and as such technology needs to adapt to account for variation among vessels and provide more consistent results. Future work will continue to investigate factors affecting the quality of the seal with emphasis on device contact surface characteristics such as the surface structure, leading to an improved understanding of the sealing process and achieving a greater quality of seal.

## Conclusion

With increasing distance from the bifurcation, there was an increase in the strength of the seal and a reduction in both vessel outer diameter and elastin content. A significant correlation was found between seal strength and both outer diameter and elastin content. However, there was also a correlation between outer diameter and elastin content making it difficult to attribute an improvement in seal strength to one factor alone. Although the vessel properties accounted for some of the variation in seal strength, substantial scatter was present in the data indicating a need for further investigation and study.

When considering the minimum threshold for a successful seal, vessels of less than 5 mm in outer diameter were shown to consistently produce a sufficient standard of seal irrespective of vessel morphology. At a distance of 64 mm of greater from the bifurcation, variation in both vessel size and vessel morphology reduced, and as a result, all seals performed in this region met the threshold of a successful seal.
